# Graphene oxide arms oncolytic measles virus for improved effectiveness of cancer therapy

**DOI:** 10.1186/s13046-019-1410-x

**Published:** 2019-09-18

**Authors:** Mao Xia, Dongjun Luo, Jie Dong, Meihong Zheng, Gang Meng, Junhua Wu, Jiwu Wei

**Affiliations:** 1 Department of Laboratory Medicine, The Affiliated Drum Tower Hospital, Medical School of Nanjing University, Nanjing, 210008 China; 2 Department of Hepatobiliary Surgery, The Affiliated Drum Tower Hospital, Medical School of Nanjing University, Nanjing, 210008 China; 3 Jiangsu Key Laboratory of Molecular Medicine, Medical School of Nanjing University, Nanjing, 210093 China; 4 The Affiliated Drum Tower Hospital, Medical School of Nanjing University, Nanjing, 210008 China

**Keywords:** Oncolytic measles virus, Delivery vector, Graphene oxide sheets, Targeted cancer therapy

## Abstract

**Background:**

Replication-competent oncolytic viruses (OVs) have been proven to be a potent anticancer weapon for clinical therapy. The preexisting neutralizing antibody in patients is a big challenge for oncolytic efficacy of OVs. Graphene oxide sheets (GOS) possess excellent biological compatibility and are easy to decorate for targeted delivery.

**Methods:**

We generated PEI-GOS-PEG-FA (Polyethyleneimine-Graphene oxide sheets-Polyethylene glycol-Folic acid). After intravenous injection, the distribution of PEI-GOS-PEG-FA in tumor-bearing mice was visualized by the IVIS Lumina XR system. Then, the oncolytic measles virus (MV-Edm) was coated with PEI-GOS-PEG-FA to form a viral-GOS complex (GOS/MV-Edm). The oncolytic effects of GOS/MV-Edm were investigated both in vitro and in vivo.

**Results:**

GOS/MV-Edm exhibited higher infectivity and enhanced oncolysis. In tumor-bearing mice, GOS/MV-Edm had significantly elevated viral replication within the tumor mass, and achieved an improved antitumor effect. Then, we confirmed that GOS/MV-Edm entered cancer cells via the folate receptor instead of CD46, a natural cognate receptor of MV-Edm. GOS/MV-Edm remained the infectivity in murine cells that lack CD46. Finally, we found that GOS/MV-Edm was effectively protected from neutralization in the presence of antiserum both in vitro and in vivo. In passively antiserum immunized tumor-bearing mice, the survival was remarkably improved with intravenous injection of GOS/MV-Edm.

**Conclusion:**

Our findings demonstrate that GOS/MV-Edm displays significantly elevated viral replication within the tumor mass, leading to an improved antitumor effect in solid tumor mouse model. Our study provided a novel strategy to arm OVs for more efficient cancer therapy. That may become a promising therapeutic strategy for cancer patients.

## Background

The attenuated measles virus, the vaccine strain Edmonston B (MV-Edm), is an oncolytic naked-stranded RNA virus that has been used in clinical trials [1]. Replicating oncolytic viruses has emerged as a promising method for the treatment of many malignancies [2, 3]. These viruses can overcome the problem of limited delivery of therapeutic agents because, in principle, the successful infection of only a few tumor cells at the initiated stage should be enough for the virus to spread among most tumor cells [4]. In animal models, MV-Edm has been shown to have oncolytic activity against human malignant glioma, lymphoma, ovarian cancer, multiple myeloma, fibrosarcoma and cutaneous T-cell lymphoma [5–9]. Moreover, a variety of replication-competent oncolytic viruses are being investigated. In particular, in October 2015, the US Food and Drug Administration (FDA) approved an oncolytic virotherapy treatment, talimogene laherparepvec (T-VEC), for patients with relapsed and unresectable melanoma [10].

As for replicating oncolytic viruses, host immune response and cellular barriers substantially limit MV-Edm infection and intratumoral spread, respectively [11]. MV-Edm is readily neutralized by serum antibodies and cleared by the human immune response. According to current virotherapy treatments, various cell carriers have been used to protect therapeutic oncolytic viruses from immune clearance and to deliver the viruses to tumor loci [6, 12, 13]. These cellular carriers include blood outgrowth endothelial cells, mesenchymal stromal cells, and osteosarcoma cells [6, 13, 14]. However, conventional cell carriers suffer from several limitations, such as clinical, logistical, immunological and ethical concerns [15, 16]. To address these limitations, researchers have sought to develop other novel oncolytic virus carriers.

Recently, nanomaterials, including microspheres, liposomes, and graphene oxides, have attracted significant attention as promising nanovehicles due to advantages in their synthesis, functional decoration, uniformity and cost-effectiveness [17–20]. Therefore, nanovehicles have been developed for the targeted delivery of many therapeutic agents, including small drug molecules, antibodies, DNA, proteins and genes [18, 21, 22].

However, unlike general agents, oncolytic viruses have distinct properties in their biological activity, have a specific size, and are sensitive to physical and chemical conditions (i.e., they are easily inactivated). These challenges and limitations have inspired further investigation of nanovehicles. Among the various nanovehicles that have been tested, graphene oxide has several outstanding properties for therapeutic delivery and biological applicability, such as high surface area, appropriate surface chemistry and number of layers, biological compatibility, easy functionalization, high purity and strong capacity in adsorption [23–25]. Sun et al. first reported that graphene oxide sheets (GOS) functionalized with antibodies could be noncovalently loaded with the cancer drug doxorubicin for selective targeting of cancer cells [26]. The researchers then applied graphene sheets for gene delivery [22, 27, 28]. In addition, due to the overexpression of folic acid (FA)-binding proteins on the surface of many types of cancer cells, FA functionalization on GOS (folic acid-GOS) is one of the most common strategies for cancer-targeting delivery [19].

In this study, to improve the targeting delivery of oncolytic viruses, nontoxic, multifunctionalized GOS with polyethylene glycol (PEG), polyethyleneimine (PEI) and FA (PEI-GOS-PEG-FA) were employed to encapsulate MV-Edm. PEG was used to increase the stability of graphene in physiological solutions; PEI was used as an adhesion promotor; and FA was used as the targeting agent. PEI-GOS-PEG-FA-decorated MV-Edm (GOS/MV-Edm) is similar to nano-Trojan horses. The encapsulation efficacy, oncolytic efficiency, tumor targeting and effectiveness in protecting against neutralizing antibodies in serum were evaluated for this type of nano-Trojan horse. That may become a novel, promising therapeutic strategy for cancer patients.

## Methods

### Cell lines and cell culture

The human lung adenocarcinoma cell lines A549 (CCL-185), HeLa (CCL-2), LLC (CRL-1642), 4 T1 (CRL-2539) and Vero African green monkey kidney cells (CCL-81) were obtained from American Type Culture Collection (ATCC, Manassas, VA). The cells were maintained in Dulbecco’s modified Eagle’s medium (DMEM) supplemented with 0.1 mM nonessential amino acids, 5% fetal bovine serum, and penicillin-streptomycin (all from Invitrogen).

### Viruses

MV-Edm and MV-Edm expressing the reporter gene luciferase (MV-Edm-Luc, kindly provided by S. Russell, Mayo Clinic, MN, USA) were propagated in Vero cells with a multiplicity of infection (MOI) of 0.02 in 2 ml OptiMEM (Invitrogen, 31,985–062) at 37 °C for 3 h. The medium was changed to DMEM supplemented with 2% FCS, and the cells were incubated at 37 °C for 1 day before being transferred to 32 °C for another day. The cells were harvested, and the viral particles were released by 2 cycles of snap freezing in liquid nitrogen and thawing in a 37 °C water bath. The viral titers were determined by 50% end-point dilution assays (TCID50) on the Vero cells.

### Reagents

The reagents used in this study are listed as follows. Graphene oxide sheets (GOS) were obtained from Nanjing XFNANO Materials Tech Co., Ltd. Six-armed PEG with a molecular weight (MW) of 10 kDa was purchased from Sunbio. Rhodamine-PEG-NHS (5kD) was purchased from Ponsure. Branched PEI with an MW of 25 kDa, *N*-(3-dimethylaminopropyl-*N*′-ethylcarbodiimide) hydrochloride (EDC), *N*-hydroxysuccinimide (NHS), 2-morpholinoethane sulfonic acid monohydrate (MES), folate (FA), dimethylformamide (DMF), N, N-diisopropylethylamine (DIPEA) and triethylamine were purchased from Sigma-Aldrich. All other chemicals were obtained from Aladdin. All solvents were used directly without further purification. All reagents were formulated as recommended by their suppliers.

### Cell viability assay

To measure viability following MV-Edm or GOS/MV-Edm infection. Cells were harvested with trypsin/EDTA and stained with trypan blue. The cell viability was determined by the trypan blue exclusion assay according to established protocols.

### Preparation of small GOS and ultrasmall carboxylated GOS

**Fig. 1:**
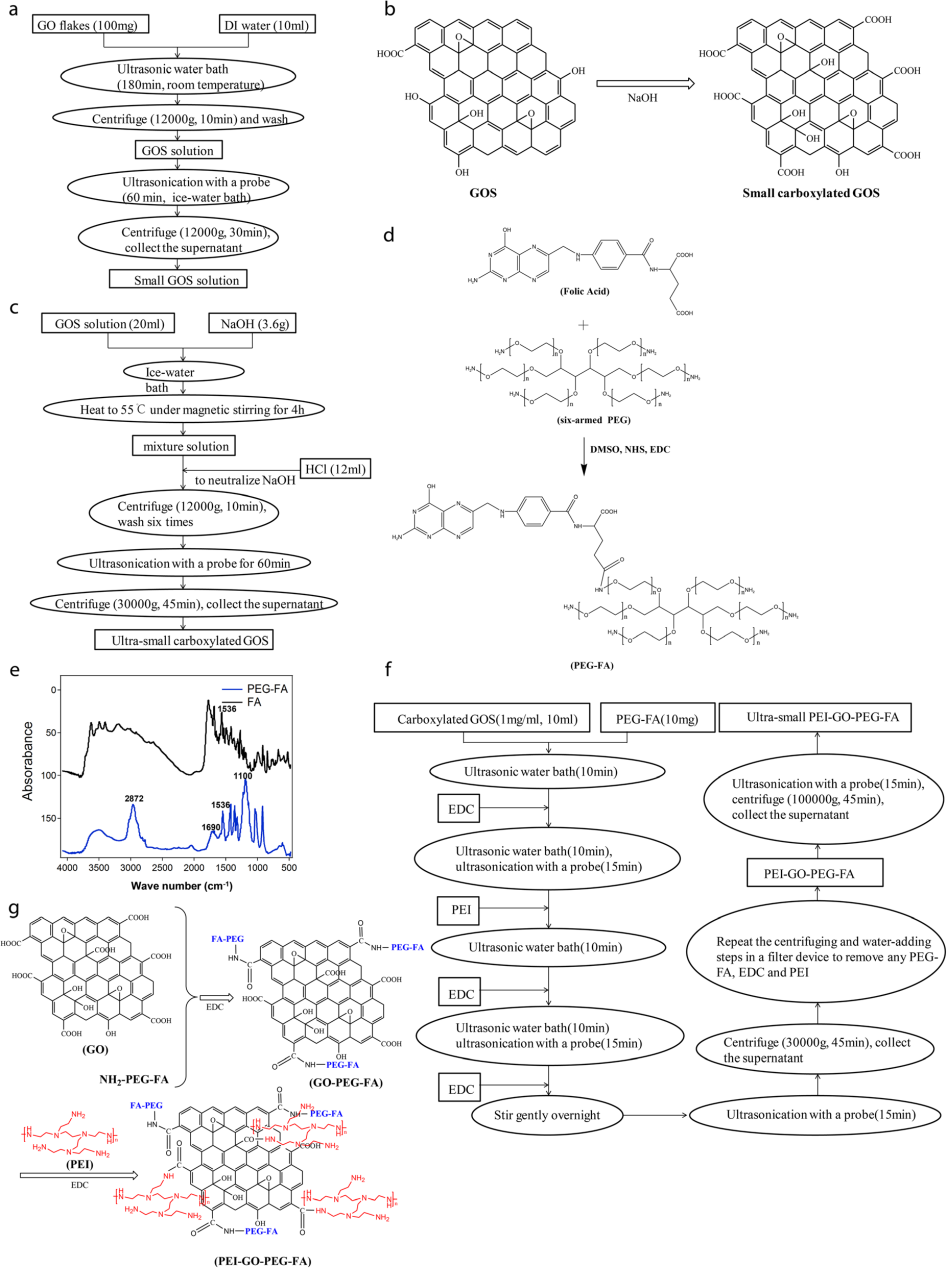
Preparation of PEI-GOS-PEG-FA. **a** The preparation process of small GOS. The rectangles represent the chemicals, and the circles represent the steps. **b** Schematic illustration of the preparation of small carboxylated GOS. The OH groups were oxidized to COOH groups on GOS by NaOH. **c** The preparation process of ultrasmall carboxylated GOS. The rectangles represent the chemicals, and the circles represent the steps. **d** Schematic illustration of the preparation of PEG-FA. Only the structure of the main product, the active carboxyl-linked conjugate, is shown. **e** FTIR spectra of FA (black) and PEG-FA (blue). Absorbance bands for PEG-FA are observed at 1690 cm^− 1^ (−NH-CO-), 2872 cm^− 1^ (C-H) and 1100 cm^− 1^ (C-O-C). In addition, a peak at approximately 1536 cm^− 1^ characteristic of FA appears in the spectrum of PEG-FA. **f** The preparation process of PEI-GO-PEG-FA solution. The rectangles represent the chemicals, and the circles represent the steps. **g** Schematic illustration of the preparation of PEI-GOS-PEG-FA. PEG-FA and PEI were both connected to GO via an amide linkage between the amino groups on PEG-FA and PEI and the carboxylic groups on GO

GO flakes (100 mg) were added to 10 ml of deionized (DI) water under vigorous stirring. The GO flakes were then dispersed in an ultrasonic water bath for 180 min at room temperature to afford a solution of GOS with a final concentration of 5 mg/ml. The mixture was then centrifuged (12, 000×g, 10 min) and washed several times with DI water. To generate small GOS, the resultant GOS solution in DI water (5 mg/ml) was treated to another round of ultrasonication with an ultrasonic probe at 400 W for 60 min in an ice-water bath, followed by centrifuging at 12,000×g for 30 min. The small GOS were obtained from the supernatant, and the supernatant was diluted with DI water to a final volume of 20 ml. The preparation process of small GOS is illustrated in Fig. 1a.

To obtain carboxylated small GOS (schematic illustration of the preparation of carboxylated GOS is shown in Fig. 1b), NaOH (3.6 g) was added in an ice-water bath to 20 ml of the small GOS solution prepared in the previous step. Then, the mixture was heated to 55 °C under magnetic stirring in an oil bath for 4 h to oxidize the OH groups to the COOH groups. HCl solution (37% (vol/vol), 12 ml) was added to the solution to neutralize the NaOH. To remove the salt, the product was washed by centrifugation at 12,000×g for 10 min, and DI water (20 ml) was added six times, followed by treatment with an ultrasonic probe at 400 W for 60 min in an ice-water bath. The product was centrifuged at 30,000×g for 45 min to generate ultrasmall carboxylated GOS in the supernatant, the concentration of which can be determined by its UV-visible NIR absorbance spectrum with a weight extinction coefficient of 47.6 mg/ml/cm at 230 nm [29]. The preparation process of ultrasmall carboxylated GOS is illustrated in Fig. 1c.

### Conjugation of FA to amine-capped polyethylene glycol (NH_2_-PEG-NH_2_)

As shown in Fig. 1d, FA (0.175 mmol), EDC (0.25 mmol) and NHS (0.25 mmol) were added to 50 ml of a 4:1 mixture of water and dimethylsulfoxide (DMSO) in the presence of 10 mM MES. After vigorous agitation and sonication for 10 min, the reaction mixture was agitated overnight. Then, 100 μl of triethylamine and 0.2526 mmol of the six-armed PEG-NH_2_ were added to the reaction mixture, and it was stirred for an additional 24 h at room temperature. After the reaction, the small-MW compounds were removed from the solution by a dialysis bag (MWCO = 2 kDa). The solution was condensed with an Amicon centrifugal filter device (Millipore, UFC910024, Billerica, MA). The PEG-FA product was obtained by lyophilization. The structure of PEG-FA was confirmed by Fourier transform infrared spectroscopy (FTIR) (Fig. 1e). Absorbance bands for PEG-FA were observed at 1690 cm^− 1^ (−NH-CO-), 2872 cm^− 1^ (C-H) and 1100 cm^− 1^ (C-O-C). In addition, a peak at approximately 1536 cm^− 1^ characteristic of FA, appeared in the spectrum, which suggests that FA was successfully bound to the PEG.

### Preparation of an ultrasmall PEI-GOS-PEG-FA conjugate

PEG-FA (10 mg) was added to the solution of ultrasmall carboxylated GOS (1 mg/ml, 10 ml) under ultrasonication in a bath sonicator at room temperature for 10 min. EDC (10 mg) was added to the mixture, and the mixture was ultrasonicated for an additional 10 min, followed by treatment with an ultrasonic probe at 400 W for 15 min in an ice-water bath. After being stirred gently at room temperature for 10 min, 50 mg of PEI in 1 ml of water was added to 10 ml of the GOS mixture, and the mixture was ultrasonicated in a bath sonicator for 10 min. Then, 20 mg of EDC was added, followed by another 10 min of ultrasonication in a bath sonicator and then treatment with an ultrasonic probe at 400 W for 15 min in an ice-water bath. After the addition of the second batch of EDC (10 mg), the reaction solution was stirred gently overnight at room temperature. After treatment with an ultrasonic probe for 15 min in an ice-water bath, the solution was centrifuged at 30,000×g for 45 min to remove any aggregates. The supernatant was collected and transferred to a 50-ml Amicon centrifugal filter device (MWCO = 100 kDa). The device was centrifuged at 2000×g for 10 min. The volume of solution remaining in the filter was less than 1 ml. The filter device was then filled with 10 ml of DI water, and the solution in the device was washed six times by repeating the centrifuging and water-adding steps to completely remove any PEG-FA, EDC and PEI in the obtained PEI-GOS-PEG-FA solution. After the final washing step, the obtained PEI-GOS-PEG-FA solution was treated with an ultrasonic probe for 15 min in an ice-water bath, and the solution was then centrifuged at 100,000×g for 45 min. The solution of ultrasmall PEI-GOS-PEG-FA was obtained by collecting the supernatant. The preparation process of the PEI-GOS-PEG-FA solution and its schematic illustration are shown in Fig. 1f and g, respectively. The concentration of PEI-GOS-PEG-FA solution was determined by analyzing the UV-visible NIR absorbance spectrum (47.6 mg ml^− 1^ cm^− 1^ at 230 nm). Unless otherwise stated, the concentration of PEI-GOS-PEG-FA used in the experiment was 1 mg/ml.

### Conjugation of rhodamine to PEI-GOS-PEG-FA

The PEI-GOS-PEG-FA solid product was obtained by lyophilization from the solution prepared in the previous step. PEI-GOS-PEG-FA (20 mg), rhodamine (1 mg) and DIPEA (0.2 μl) were added to 0.5 ml of DMF under dark conditions. After vigorous agitation, the reaction mixture was stirred gently overnight at room temperature in the dark. After sonication in a bath sonicator at room temperature for 1 h, the small-MW compounds were removed from the reaction solution by a dialysis tubing (MWCO = 10 kDa). Finally, PEI-GOS-PEG-FA labeled with the fluorescent dye rhodamine was obtained in the solution [30].

### Quantitative RT-PCR

For quantitative RT-PCR (qPCR), total cellular RNA was extracted with TRIzol (Invitrogen, 15,596–026), and 1 μg of RNA was reverse-transcribed using the synthesis system (TaKaRa, DRR036A). qPCR was performed using the real-time PCR system (ABI 7300). Gene expression was calculated with the comparative Ct method and normalized to the endogenous levels of GAPDH. The primer sequences used for qPCR are listed in Table 1.

**Table 1:** Primers

	5′-primer 1	3′-primer 2
GAPDH	CCACCCATGGCAAATTCCATGGCA	TCTAGACGGCAGGTCAGGTCCACC
MV-Edm N-protein	ACATTAGCATCTGAACTCGGTATCAC	TTTTCGCTTTGATCACCGTGTA

### Luciferase assay

The luciferase activity of cells infected with MV-Edm expressing luciferase (MV-Edm-Luc) was monitored based on their luminescence following the addition of luciferin (Gold Biotechnology, St. Louis, MO, LUCK-1G) at a concentration of 150 μg/ml according to the instructions of the manufacturer.

### Animal studies

Immunodeficient nude mice ((Foxn1nu) mut/mut male, 5–6 weeks old) obtained from the Model Animal Research Center of Nanjing University (Nanjing, China) were used for in vivo assays. All animal work was approved by the Animal Care Committee of Nanjing University and was carried out in accordance with Institutional Animal Care and Use Committee guidelines. The mice were injected subcutaneously with 5 × 10^6^ HeLa cells/0.1 ml/mouse in the left flank. On the 10th day, the tumors became visible. At that point, the mice were randomly assigned to different groups. The tumor volume was calculated using the following formula: 0.5236 × L1 (L2)^2^, where L1 is the long diameter and L2 is the short diameter. Tumors were measured once every 3 days, and their survival was monitored daily. Mice exhibiting moribund behavior were euthanized.

### Transfer of antibodies against measles virus to mice

Serum was isolated from a healthy donor with immunity to measles, inactivated at 56 °C for 30 min and centrifuged at 4000×g. To neutralize the MV-Edm in vivo, a group of mice bearing HeLa tumors was immunized by intraperitoneal injection of 500 μl of human measles immune serum 18 h prior to injection of either MV-Edm or GOS/MV-Edm.

### Visualization of MV-Edm replication in vivo

Fourteen days after injection of the HeLa cells, once the tumors were established, the mice received injections of MV-Edm or PEI-GOS-PEG-FA/MV-Edm (GOS/ MV-Edm). Three days after this injection, the mice were anesthetized and injected intraperitoneally with D-luciferin (Gold Biotechnology, St. Louis, MO, LUCK-1G) and subjected to a luciferase assay using the IVIS Lumina XR system (Caliper Life Sciences, Hopkinton, MA). The level of firefly luciferase was expressed in terms of the ROI value, which was normalized to tumor volume.

### Statistical analysis

Results are expressed as the mean ± standard error of the mean (SEM). Student’s t tests were used for statistical analyses. Survival data were compared using the log-rank test. *P*-values less than 0.05 were considered significant.

## Results

### Characterization of ultrasmall PEI-GOS-PEG-FA and its tumor-targeting capacity

**Fig. 2:**
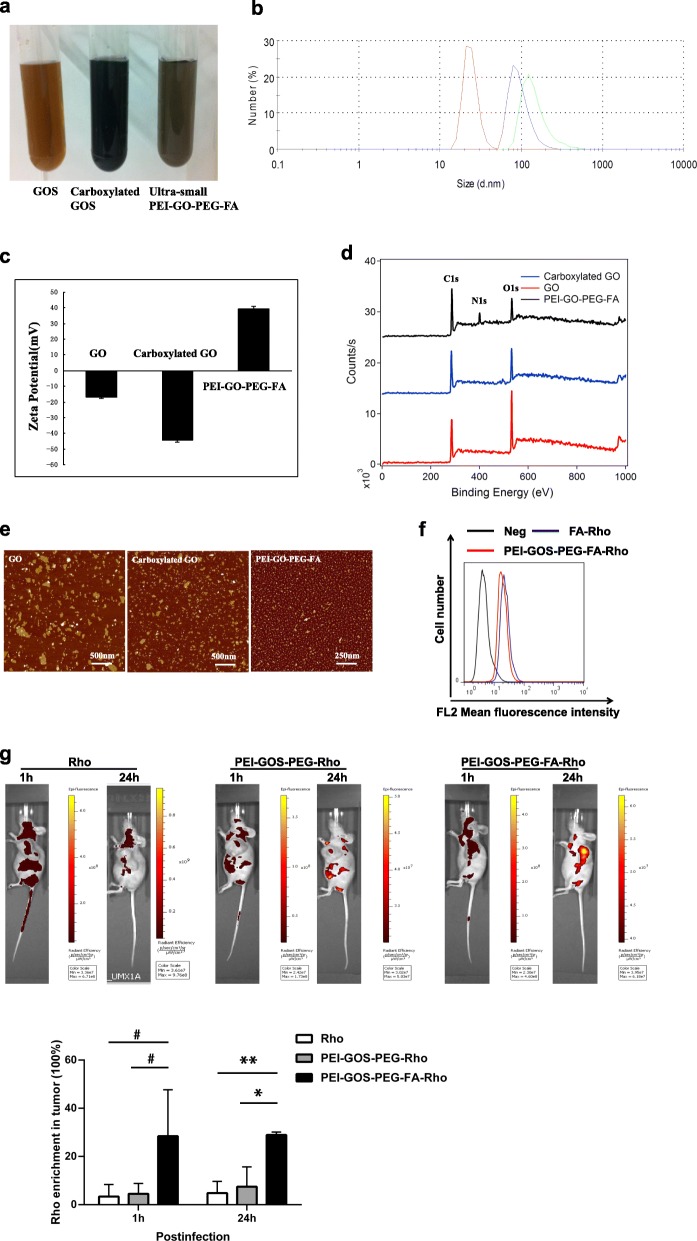
Characterization of ultrasmall PEI-GOS-PEG-FA and its tumor-targeting effect. **a** Photographs of GOS, carboxylated GOS and PEI-GO-PEG-FA in DI water show a visible color difference. **b** Representative diagram from Zetasizer Nano ZSP for the sizes of various GOS derivatives. **c** Zeta potential values of various GO derivatives. **d** XPS binding energy survey of the PEI-GO-PEG-FA conjugate (black), GOS (red) and carboxylated GOS (blue). **e** AFM images of small GOS, ultrasmall carboxylated GOS and PEI-GO-PEG-FA. **f** HeLa human cervical carcinoma cells largely express FR. FA and PEI-GOS-PEG-FA were labeled with rhodamine (FA-Rho and PEI-GOS-PEG-FA-Rho), a commonly used fluorescent dye. Cell-surface FR expression of the HeLa cells was determined by fluorescence-activated cell sorting analysis. **g** A total of 5 × 10^6^ HeLa cells/0.1 ml/mouse was subcutaneously injected into the left flanks of 5- to 6-week-old male Foxn1nu mut/mut mice; 10 days later, the tumors became visible. The mice were randomly divided into three groups. PEI-GOS-PEG or PEI-GOS-PEG-FA was labeled with rhodamine; then, nude mice bearing HeLa human cervical carcinoma tumors were intravenously injected with Rho, PEI-GOS-PEG-Rho or PEI-GOS-PEG-FA-Rho (200 μl of a 0.15 mg/ml solution for each mouse; a dose of 20 mg/kg) and then spectrally imaged by the IVIS Lumina XR system at 1 h and 24 h postinjection. The percentage of tumor enriched Rho was calculated by dividing the fluorence intensity in tumor by total (lower panel, *n* = 3). * *p* < 0.05, **p < 0.01

GOS, carboxylated GOS and ultrasmall PEI-GOS-PEG-FA conjugates are all highly stable in DI water (Fig. 2a). The size and ζ-potential that signifies the electrophoretic mobility of the charged surface of the GOS, carboxylated GOS and PEI-GOS-PEG-FA were examined using a Zetasizer Nano ZSP instrument (Malvern, UK) [31]. The size of the small GOS was 153.1 ± 7.2 nm and that of the ultrasmall carboxylated GOS was 94.1 ± 4.2 nm. As expected, the ultrasmall PEI-GOS-PEG-FA conjugate was only 25.4 ± 4.2 nm (Fig. 2b and Table 2). GOS showed a negative zeta potential of − 16.7 ± 0.9 mV, while the zeta potential of carboxylated GOS was − 44.2 ± 1.5 mV. However, the PEI-GOS-PEG-FA conjugate showed a high positive zeta potential, indicating that a considerable change in the surface charge of GOS occurred due to PEI conjugation (Fig. 2c and Table 3). X-ray photoelectron spectroscopy (XPS) was used to verify the immobilization of PEI on the GOS. As expected, an intense N-binding peak at 400 eV corresponding to the N 1s_1/2_ orbital was observed only in the PEI-GOS-PEG-FA conjugate and not in the GOS and carboxylated GOS, confirming the presence of PEI on the GOS (Fig. 2d) [32]. Atomic force microscopy (AFM) images (Fig. 2e) showed that the size of the PEI-GOS-PEG-FA sheets was much smaller than that of the starting GOS sample due to the ultrasonication that cleaved the graphene sheets.

**Table 2:** The sizes of various GO derivatives

	Diameter (nm) Mean ± s. d.
GO	153.1 ± 7.2
Carboxylated GO	94.1 ± 4.2
PEI-GO-PEG-FA	25.4 ± 4.2

**Table 3:** Zeta potential values of various GO derivatives

	Zeta Potential (mV) Mean ± s. d.
GO	−16.7 ± 0.9
Carboxylated GO	−44.2 ± 1.5
PEI-GO-PEG-FA	39.4 ± 1.5

To study the behaviors and biodistribution of PEI-GOS-PEG-FA in vivo, we labeled PEI-GOS-PEG and PEI-GOS-PEG-FA with rhodamine (PEI-GOS-PEG-Rho and PEI-GOS-PEG-FA-Rho, respectively), a commonly used fluorescent dye. This strategy provides a reliable and convenient method for the real-time and in situ monitoring of animal models. Nude mice bearing human cervical carcinoma HeLa cells were intravenously injected with PEI-GOS-PEG-FA-Rho and then spectrally imaged by an IVIS Lumina XR system. Most HeLa human cervical carcinoma cells expressed folate receptor (FR) (Fig. 2f). As shown in Fig. 2g, PEI-GOS-PEG-FA-Rho was widely dispersed in the whole body of a mouse in the first 60 min postinjection (p.i.) and became enriched in the tumor over time. Prominent uptake of PEI-GOS-PEG-FA-Rho was observed in the tumor, while relatively low signals were observed in other parts of the mouse body at 24 h p.i. Mice injected with PEI-GOS-PEG-Rho or free Rho solutions at the same concentrations showed relatively lower fluorescent signaling intensity. These results suggest that PEI-GOS-PEG-FA-Rho has efficient tumor-targeting effects; in contrast, the results of the PEI-GOS-PEG-Rho and free Rho tests show limited tumor uptake in the absence of the targeting ligand.

### MV-Edm can be effectively encapsulated in graphene oxide

**Fig. 3:**
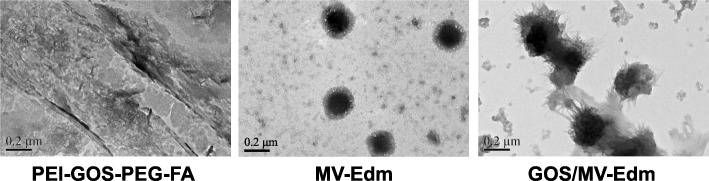
Encapsulation of MV-Edm in GOS. **a** Encapsulation of MV-Edm in PEI-GOS-PEG-FA was observed by transmission electron microscopy (TEM). Similar results were obtained in three independent experiments. Scale bars, 200 nm

To evaluate the possibility of PEI-GOS-PEG-Rho for use as tumor-targeting carriers for MV-Edm, we first verified whether PEI-GOS-PEG-Rho can encapsulate MV-Edm. Using transmission electron microscopy, we observed that PEI-GOS-PEG-Rho was capable of encapsulating MV-Edm (Fig. 3). Furthermore, the size and zeta potential of MV-Edm, PEI-GOS-PEG-FA and GOS/MV-Edm were measured by XPS at 10 μl MV-Edm (10^8^ TCID50/ml) and GOS (0.15 mg/ml); the ratio is v/v. The size and zeta potential of GOS/MV-Edm were larger than those of unbound MV-Edm or PEI-GOS-PEG-Rho and were ratio dependent (Table 4). These results imply that the as-prepared PEI-GOS-PEG-Rho successfully capsulated MV-Edm; MV-Edm capsulated by PEI-GOS-PEG-FA is identified as GOS/MV-Edm.

**Table 4:** Characterization of MV-Edm encapsulation in GOS

				PEI-GOS-PEG-FA: MV-Edm(v/v ratio)		
	PEI-GOS- -PEG-FA	MV-Edm	2:1	3:1	4:1	5:1
Size (nm)	48.09	241.4	271.2	290.8	1164	1154
Zeta (mV)	40.2	−21.2	−10.2	−9.9	−2.43	6.57

### GOS/MV-Edm exhibits a more effective infectious and oncolytic capacity than MV-Edm in cancer cells

**Fig. 4:**
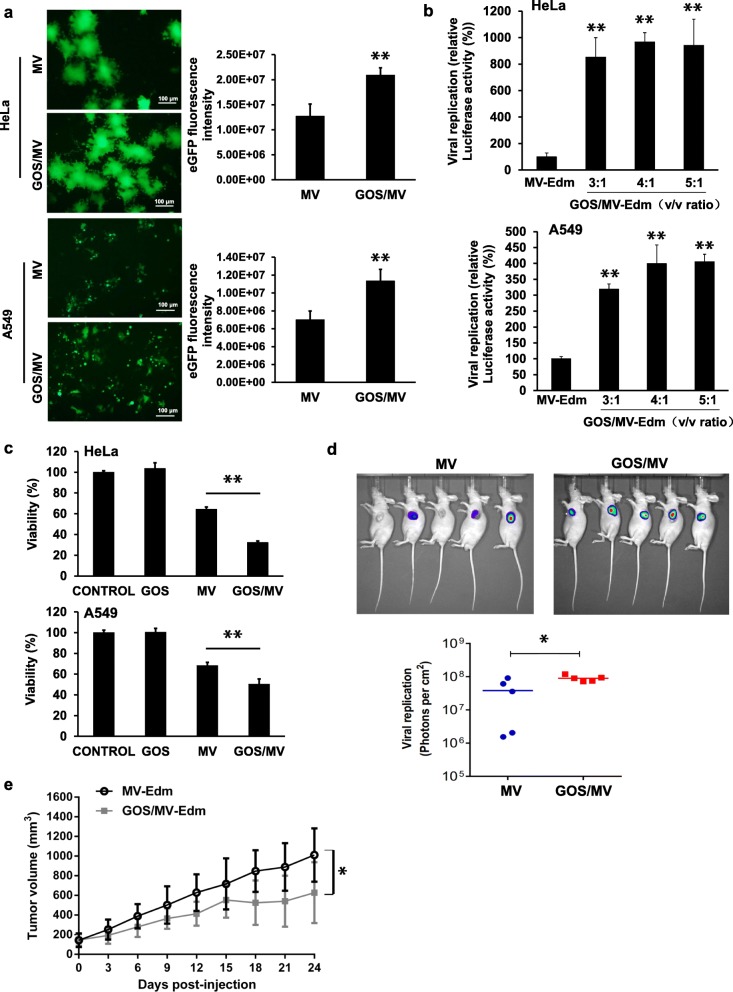
Encapsulation of MV-Edm in GOS promoted viral infection and improved oncolytic effects, and intratumorally injected GOS/MV-Edm significantly inhibited the tumor growth of mice in vivo. **a** HeLa and A549 cells were infected with MV-Edm-eGFP and GOS/MV-Edm-eGFP at an MOI of 0.5 and 5 μg/ml PEI-GOS-PEG-FA. The fluorescence images of the cells and the GFP fluorescence intensities were measured at 48 h postinfection. **b** The HeLa and A549 cells were infected with MV-Edm-Luc or GOS/MV-Edm-Luc at an MOI of 0.5, and the luciferase activity was monitored based on its luminescence 48 h after the virus injection. **c** Cell death was quantified by trypan blue exclusion in HeLa and A549 cells at 48 h postinfection. Similar results were obtained in three independent experiments. Mean of triplicates, # p indicates not significant, *p < 0.05, **p < 0.01. **d** A total of 5 × 10^6^ HeLa cells/0.1 ml/mouse was subcutaneously injected into the left flanks of 5- to 6-week-old male Foxn1nu mut/mut mice; 10 days later, the tumors became visible. The mice were randomized into two groups (*n* = 5 for each group). Then, two groups of mice were intratumorally injected with MV-Edm-Luc (2 × 10^6^ TCID50 per mouse). Luciferase activity was monitored by an in vivo luminescence imaging system 72 h after virus injection (left panel). Photons per cm^2^ tumor were quantified (lower panel). **e** On the first day and the fourteenth day after implantation of HeLa cells, naked MV-Edm or GOS/MV-Edm were injected peritumorally into the mice bearing HeLa tumors. The volumes of tumors treated with GOS/MV-Edm (black line) were clearly smaller than those treated with naked MV-Edm (gray line). Means are shown (*n* = 5 of each), * *p* < 0.05, **p < 0.01

Next, we investigated whether the successfully encapsulated MV-Edm (GOS/MV-Edm) retained its ability to infect cancer cells. HeLa and A549 cells strongly express the MV-Edm receptor (CD46), which allows the MV-Edm to enter the cells. In Fig. 4a, HeLa and A549 cells were successfully infected with MV-Edm using GOS/MV-Edm-eGFP. Interestingly, fluorescence microscopy showed that MV-Edm-eGFP spread more rapidly in GOS/MV-Edm-infected cells than in naked MV-Edm-infected cells. MV-Edm-Luc replicated more rapidly in GOS/MV-Edm cells than in naked MV-Edm-infected cells, as shown in Fig. 4b. Furthermore, the oncolytic effect of GOS/MV-Edm was significantly stronger than that of naked MV-Edm in HeLa or A549 cells (Fig. 4c).

To confirm the antitumor effects of GOS/MV-Edm in vivo, we employed a nude murine model burdened with HeLa human cervical cancer cells infected with either GOS/MV-Edm or naked MV-Edm. On days 1 and 14 postinoculation of the HeLa cells, mice bearing HeLa tumors were treated with naked MV-Edm or GOS/MV-Edm. GOS/MV-Edm was more efficient in replicating than naked MV-Edm in vivo (Fig. 4d). The tumor volumes observed in the two groups were significantly different. The volumes of the tumors in mice treated with GOS/MV-Edm were clearly smaller than those of the tumors treated with naked MV-Edm (Fig. 4e). These results indicate that MV-Edm encapsulated in PEI-GOS-PEG-FA has a significantly more impactful oncolytic effect than naked MV-Edm in vitro and in vivo.

### PEI-GOS-PEG-FA encapsulated MV-Edm infects cells via the folate receptor

**Fig. 5:**
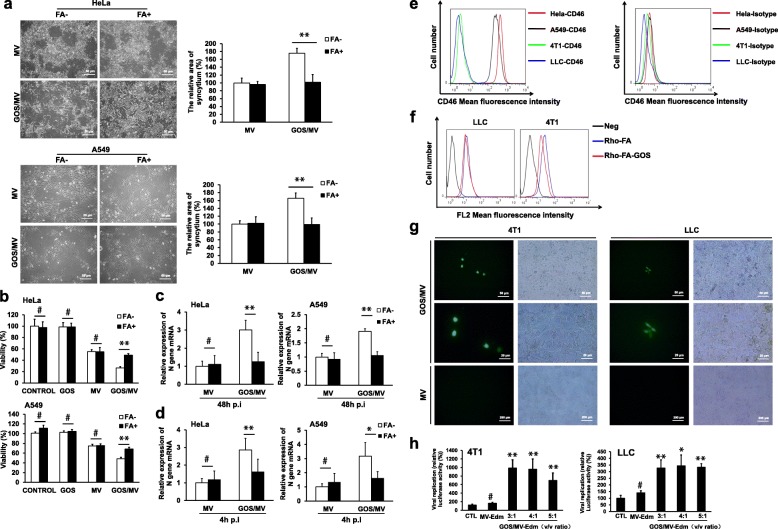
Folate targeting/retargeting of GOS/MV-Edm. HeLa and A549 cells were infected with naked MV-Edm or GOS/MV-Edm in FA^−^ DMEM (DMEM with FA depleted from the cell medium) or FA^+^ DMEM (DMEM with abundant FA (10 mM) to give only a few available free FRs on the cell surfaces). **a** Syncytium formation was observed by phase-contrast microscopy (left panel) and further analyzed using ImageJ software (right panel) 48 h postinfection. **b** The oncolytic effect (cell death) was quantified by trypan blue exclusion in cells at 48 h postinfection in FA^−^ or FA^+^ medium. The expression of N viral structural genes was quantified by qRT-PCR in cells 48 h postinfection **c** or 4 h postinfection **d**. **e** CD46 expression on the surfaces of human cells (HeLa and A549 cells) and murine cells (4 T1 and LLC cells) was determined by flowcytometry using anti-CD46 and isotype-matched control antibodies, respectively. **f** The surface expression of FR in murine cells (4 T1 and LLC cells) was determined by fluorescence-activated cell sorting analysis using Rho-FA and Rho-FA-GOS. **G** Fluorescence images of cells 60 h postinfection. GOS/MV-Edm-eGFP infected the murine cells (4 T1 and LLC cells), but the naked MV-Edm absolutely could not infect the murine cells. **h** The murine cells (4 T1 and LLC cells) were infected with MV-Edm-Luc or GOS/MV-Edm-Luc (10^8^ TCID50/ml) at an MOI of 1 and 0.15 mg/ml GOS, and the ratio is v/v. Luciferase activity was monitored by a luminescence system 48 h after virus infection. Mean of triplicates, #p indicates not significant, *p < 0.05, **p < 0.01

FR is overexpressed in many cancer cells, which makes folate an attractive target for cancer therapies. HeLa and A549 cells positively express FR [33, 34]. To explore whether GOS/MV-Edm enabled the virus to enter the cells via the FR, HeLa and A549 cells were infected with GOS/MV-Edm or naked MV-Edm in FA-free DMEM or DMEM with high levels of FA (Fig. 5a). FA-free medium (FA^−^) was used to ensure the availability of FR on the surface of the FR-expressing cancer cells, and DMEM with abundant FA (FA^+^) was used to block the majority of the FRs on the cancer cell surfaces [35]. For naked MV-Edm infection, the cells in the FA^+^ or FA^−^ medium displayed almost identical syncytia and oncolytic effects. However, the syncytia and oncolytic effects of GOS/MV-Edm in the FA^−^ medium were significantly increased compared with those in the FA^+^ group (Fig. 5a and b). The expression of N viral structural genes was quantified by qRT-PCR in HeLa and A549 cells infected with naked MV-Edm or GOS/MV-Edm in FA^+^ or FA^−^ medium (Fig. 5c and d). These data confirm that the facilitating effect of PEI-GOS-PEG-FA on MV-Edm to enter cancer cells is dependent on the FR.

To determine whether GOS/MV-Edm enabled MV-Edm to enter into the cells by the FR rather than the MV-Edm receptor (CD46), we studied whether GOS/MV-Edm could infect murine cells that lack the MV-Edm receptor (CD46) and have an abundance of the FA receptor. Indeed, the murine cells (4 T1 and LLC cells) almost did not express CD46, while the human cells (HeLa and A549 cells) strongly expressed this molecule (Fig. 5e). In addition to expressing CD46, 4 T1 and LLC cells express a certain amount of FR (Fig. 5f). Interestingly, observation from fluorescence microscopy showed that GOS/MV-Edm-eGFP, rather than the naked MV-Edm, could infect the murine 4 T1 and LLC cells but the naked MV-Edm could not (Fig. 5g). In addition, after entering cancer cells that were facilitated by PEI-GOS-PEG-FA, MV-Edm could replicate in the murine cells, which express a certain amount of FR (Fig. 5h). However, this phenomenon was not observed in naked MV-Edm-infected murine cells. These data show that PEI-GOS-PEG-FA enables MV-Edm to enter the cells through the FR.

### GOS/MV-Edm remains infective in the presence of antimeasles neutralizing serum

**Fig. 6:**
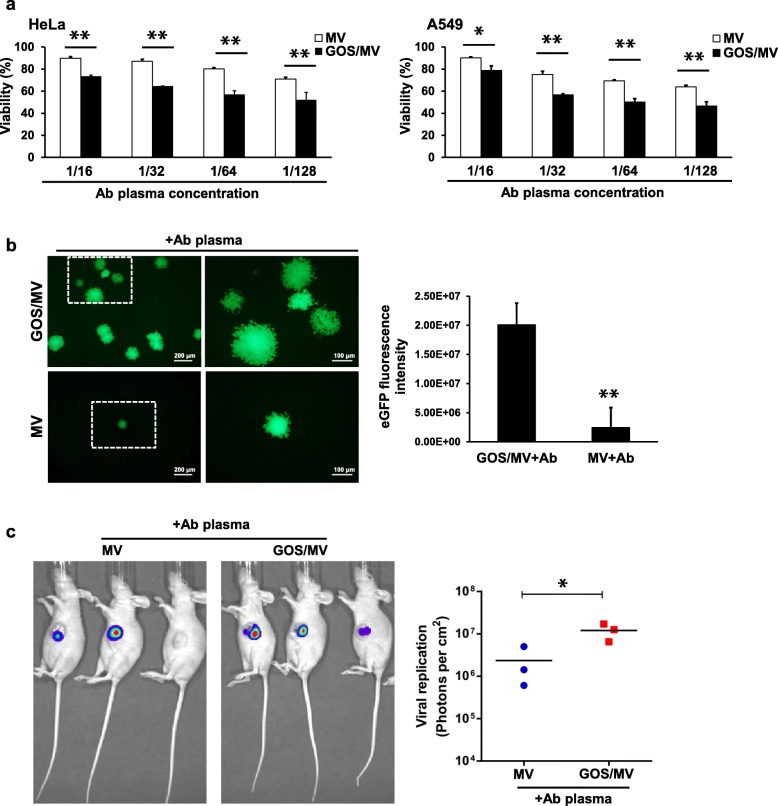
Neutralization assay. **a** MV-Edm and GOS/MV-Edm were incubated with human MV immune serum (Ab) and added to HeLa or A549 cells at an MOI of 0.5. The plasma with a high neutralizing antibody titer was diluted to 1/16, followed by one-half dilutions up to 1/128. The oncolytic effect (cell death) was quantified by trypan blue exclusion in cells at 48 h postinfection. **b** MV-Edm-eGFP and GOS/MV-Edm-eGFP were incubated with human MV immune serum and added to HeLa cells at an MOI of 0.5. The fluorescence images of cells 60 h postinfection and GFP fluorescence intensity of three images were measured at 60 h postinfection. Mean of triplicates, # p indicates not significant, *p < 0.05, **p < 0.01. **c** The nude mice bearing HeLa human cervical carcinoma tumors were randomized into two groups (*n* = 3 for each group). On the first and fourteenth days after injection of the HeLa cells, naked MV-Edm-Luc or GOS/MV-Edm-Luc were injected peritumorally into the mice bearing HeLa tumors, and the mice had been immunized by intraperitoneal injection of 500 μl human immune serum with measles antibodies 18 h prior to injection of naked MV-Edm-Luc or GOS/MV-Edm-Luc. Two groups of mice were injected with MV-Edm-Luc or GOS/MV-Edm-Luc (2 × 10^6^ TCID50 per mouse) via the tail vein. Luciferase activity was monitored by an in vivo luminescence imaging system 72 h after virus injection (left panel). Photons per cm^2^ of tumor were quantified (right panel). Means are shown (*n* = 3 of each), * *p* < 0.05, **p < 0.01

The clinical application of MV-Edm as an oncolytic virus is limited by the neutralization of preexisting measles antibodies in most patients due to previous immunization or prior oncolytic viral therapy [16, 36]. To investigate whether the GOS could protect MV-Edm from neutralization by human measles antibodies, plasma with a high titer of neutralizing antibodies was diluted 16 times, followed by serial one-half dilutions up to 1:128. The serum with a high titer of neutralizing antibodies was obtained from a healthy donor with immunity against measles as defined by high levels of measles antibodies using the PRN assay [37]. As expected, MV-Edm infection towards HeLa and A549 cells was completely blocked when undiluted human immune MV serum was used. After incubation with the neutralizing plasma, MV-Edm encapsulated in PEI-GOS-PEG-FA had a markedly stronger oncolytic effect than the naked MV-Edm incubated with the neutralizing plasma (Fig. 6a). Furthermore, fluorescence microscopy showed that MV-Edm-eGFP replicated more rapidly in GOS/MV-Edm cells than in naked MV-Edm-infected cells following incubation with the neutralizing antibody (Fig. 6b). These results suggest that GOS/MV-Edm is more resistant to neutralizing antibodies and that GOS/MV-Edm is capable of infecting cancer cells despite continuous exposure to human immune serum containing measles antibodies.

In addition, the in vivo data show that GOS/MV-Edm was more efficient in targeting the HeLa tumor than naked MV-Edm in the presence of antimeasles virus antibodies (Fig. 6c).

### GOS/MV-Edm significantly improves the antitumor efficacy in antiserum passively immunized tumor-bearing mice

**Fig. 7:**
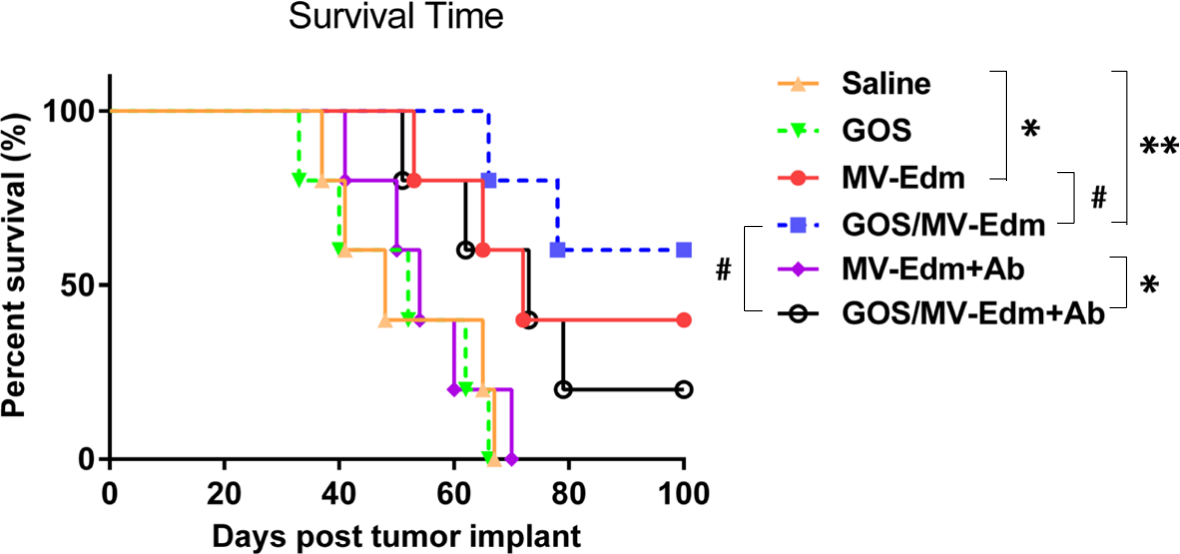
MV-Edm protection by GOS in vivo. The nude mice bearing HeLa human cervical carcinoma tumors were randomized into five groups (*n* = 5 for each group). On day 1 and 14 after inoculation of HeLa cells, mice received peritoneal injection of 500 μl human immune serum containing measles antibodies for 18 h followed by intravenous injection of either naked MV-Edm (purple line) or GOS/MV-Edm (black line). Or mice received the same volume of saline for 18 h followed by intravenous injection of either naked MV-Edm (red line) or GOS/MV-Edm (blue line). The mice treated with saline alone (orange line) or PEI-GOS-PEG-FA alone (green line) were used as controls. Survival of the mice was determined and plotted for survival analysis. Mice were euthanized when the tumor volume reached to 2 cm3 or when they experienced intolerable suffering. All mice surviving at day 100 were euthanized at the end of the study. Statistical testing was performed by log-rank test. # p indicates not significant, *p < 0.05, **p < 0.01

Next, to elucidate whether the resistance of GOS/MV-Edm to neutralizing antibodies could be observed in vivo, we evaluated the in vivo anticancer effects of GOS/MV-Edm and naked MV-Edm against HeLa human cervical cancer cells using a tumor-bearing nude mouse model recombined with human serum containing antimeasles antibodies. On the first and fourteenth days after inoculation of the HeLa cells, either naked MV-Edm or GOS/MV-Edm was intravenously injected into the HeLa tumor-burdened mice in the presence or absence of 500 μl of human immune serum containing measles antibodies. Mice received GOS/MV-Edm displayed a slightly prolonged survival compared with those treated with MV-Edm, although no significant difference reached. However, GOS/MV-Edm injection led to a significant survival benefit for HeLa tumor-bearing mice compared with those receiving MV-Edm in the presence of the MV-neutralizing serum antibodies (Fig. 7). To be noted, the antitumor efficacy of naked MV-Edm was completely abrogated when mice were administrated with MV-neutralizing antibodies. In the contrast, the GOS/MV-Edm remained its capability to prolong the mice survival in the presence of neutralizing antibodies. These data reveal that GOS/MV-Edm is more resistant to the neutralizing antibody and displays a higher therapeutic effect in vivo than naked MV-Edm.

## Discussion

Preexisting virus-neutralizing antibodies and inefficient tumor-targeted delivery are major hurdles in the road to improving the oncolytic effect of viral therapies on malignant carcinomas [36, 38–40]. In this study, we showed that replicating oncolytic MV-Edm capsulated within PEI-GOS-PEG-FA may be a promising approach to overcoming these hurdles. The present study has shown that the GOS/MV-Edm complexes led to enhanced infecting efficiency, increased resistance to neutralizing measles antibodies in human serum in a concentration-dependent manner, and elevated efficiency of targeted delivery. Subsequently, GOS/MV-Edm treatment displayed elevated antitumor effects in vivo in terms of reduced tumor volume and prolonged the life of tumor-bearing mice. The in vivo efficacy of GOS/MV-Edm is mainly based on active targeting, retention effects and permeability [41]. Fortunately, the in vivo data showed encouraging results when the treatment was translated from in vitro to in vivo. In addition, no significant GOS-related side effects were observed in this study.

For the utilization of oncolytic viruses in the clinical treatment of cancer, it would be difficult for viruses that were delivered systemically to reach the tumor sites. Despite the promising preclinical results of MV-Edm as a selective cancer therapy, MV-Edm has shown oncolytic efficacy only for patients without antimeasles virus antibodies in clinical trials [42]. Intravenous administration has been shown to result in sequestration due to macrophage uptake and preexisting neutralizing antibodies [13, 16]. Therefore, various carriers are promising agents with considerable potential as oncolytic therapeutics. Previously, cell carriers were designed to solve these problems by protecting oncolytic viruses from immune clearance and delivering viruses to tumor loci [13, 16]. Cell carriers have inherent tumor tropism by which they could deliver the oncolytic virus more specifically to tumor sites. Thus, the specifically designed cell carriers need to be designed for each person rather than a general population; otherwise, they could elicit immune rejection because of “nonself” components [43]. One phase I clinical trial for ovarian cancer used autologous/patient-derived mesenchymal stem cells as oncolytic measles virus carriers [44]. These challenges would cause the treatment to be unaffordable for the general public and significantly prolong the time of therapy. In addition, traditional cell carriers suffer from several other drawbacks, such as logistical, immunological and ethical considerations [44]. Recently, an in vitro method for the liposomal encapsulation of an oncolytic adenovirus was developed, and the encapsulated viruses remained able to infect cancer cells. Although this study remains restricted to in vitro data and its effect in vivo remains unclear, this novel delivery strategy has potential for oncolytic therapy [33]. Compared to traditional cell carriers, these nanovehicles have many unique advantages, such as mass synthesis, functional decoration, multiple detection, and cost-effectiveness [41]. In this study, complete PEI-GOS-PEG-FA encapsulation reduced the risk of adverse immune responses when the virus was administered at a high dose, and the encapsulation reduced clearance from the bloodstream due to neutralizing antibodies. In addition, the increased infection efficiency of GOS/MV-Edm caused a stronger antitumor effect, which eventually prolonged the lives of tumor-bearing mice. Our investigations regarding the dynamics of circulation and tumor accumulation after intratumoral or intravenous administration of GOS/MV-Edm assess the efficacy of the platform in vivo well. A better comprehension of the cellular release of the MV-Edm from the GOS capsulation may allow the development of an improved oncolytic virus delivery platform. Generally, virus release in a cell may be due to changes in the environmental conditions between the extracellular matrix and the cytoplasm.

On the other hand, the naked MV-Edm could enter numerous types of tumor cells via the ubiquitously expressed protein CD46 [45]. Recently, some chimeric measles viruses were recombined with antigens for tumor-specific ligands such as CD20, CD133, and synthetic microRNA for tumor-targeting sequences [46, 47], however, these chimeric viruses with retargeted tropisms also raised safety concerns, including the ability of these chimeric virus mutations to impair cell entry [48] and their limited specificity for certain tumor types. Clearly, there is an urgent need to develop a generally applicable platform that can target tumor sites. In this study, GOS/MV-Edm showed specific and efficient targeting to tumor cells, which was crucial for therapeutic efficacy. GOS is a promising biological therapy carrier due to its excellent biological compatibility and clear targeting movement after decoration [41, 49]. The expression of folate receptor, folic acid (FA)-binding protein, is elevated in many types of cancer cells, and GOS functionalizd with FA is widely used as a strategy for cancer-targeting delivery. As a ligand, FA could be covalently conjugated to GOS and confer a tumor-targeted delivery [50, 51]. In our study, we developed a novel strategy to achieve both virus protection and targeted release within the tumor by encapsulating oncolytic measles virus and conjugating pegylated folate. These in vivo data showed that GOS/MV-Edm had effective and targeted oncolytic properties even when exposed to neutralizing antibodies. However, the exact release process of MV-Edm from GOS is still unclear. Further investigations regarding the release mechanism and stability of GOS/MV-Edm in blood circulation are needed to elucidate the efficacy of the strategy in vivo.

## Conclusions

**Scheme 1:**
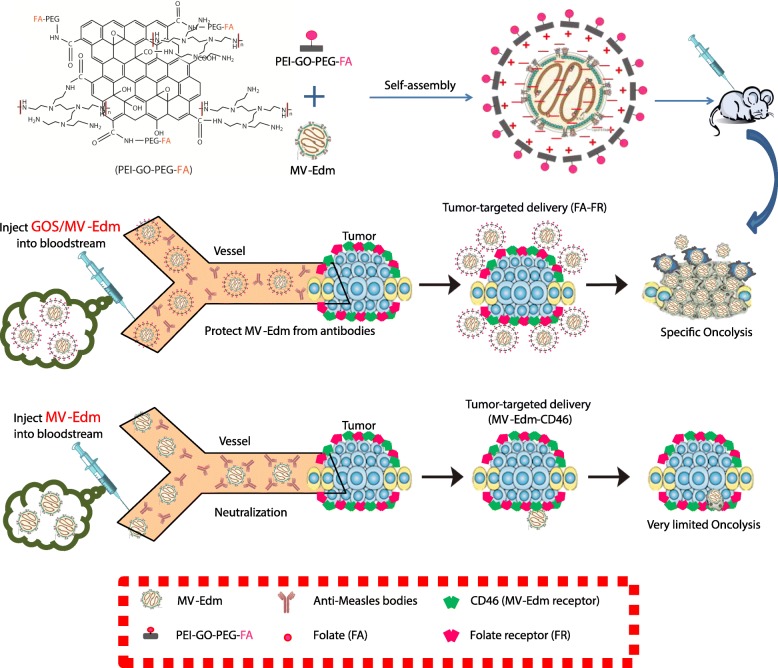
Schematic illustration for the GOS/MV-Edm and the Graphene oxide arms oncolytic measles virus for more efficient cancer therapy than naked MV-Edm

In this study, it was shown that the encapsulation of oncolytic MV-Edm within PEI-GOS-PEG-FA (GOS/MV-Edm) has the potential to improve the oncolytic effect of viral therapies on malignant carcinomas by protecting viruses from neutralization by antivirus antibodies in human blood, extending the circulation time and improving the ability of tumor targeting (Scheme. 1). Our current work exploits the fact that GOS/MV-Edm was more resistant to neutralizing antibodies and displayed a higher therapeutic effect in vivo than naked MV-Edm. In summary, a novel, nontoxic encapsulation method was developed to enhance the targeted delivery of MV-Edm to cancer cells even with antivirus antibodies. These findings could stimulate preclinical and clinical studies that will explore the therapeutic aspects of this strategy.

## Data Availability

All data analyzed during this study are included in this manuscript.

## Abbreviations

DIPEA: N, N-diisopropylethylamine
DMF: Dimethylformamide
EDC: N-(3-dimethylaminopropyl-N′-ethylcarbodiimide) hydrochloride
FA: Folic acid
FTIR: Fourier transform infrared spectroscopy
GOS: Graphene oxide sheets
MES: 2-morpholinoethane sulfonic acid monohydrate
MV-Edm: Attenuated measles virus of the Edmonston strain
NHS: N-hydroxysuccinimide
OVs: Oncolytic viruses
PEG: Polyethylene glycol
PEI: Polyethyleneimine
